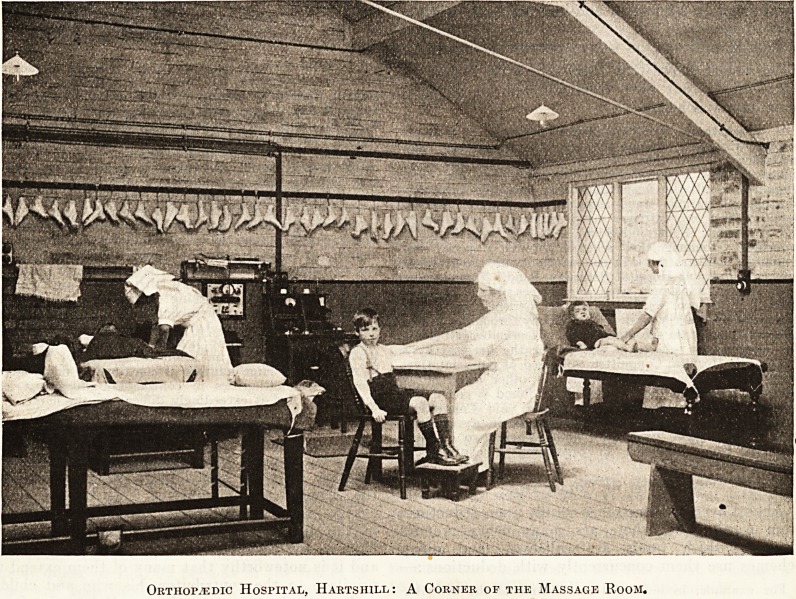# The Hartshill Orthopædic Hospital

**Published:** 1923-10

**Authors:** 


### HARTSHILL ORTHOPÆDIC HOSPITAL.

THE North Staffordshire Cripples' Aid Society for the first time since 1918 is able to state that its finances are on a sound footing. This is due mainly to an extraordinarily successful bazaar held at Stoke-on-Trent, which benefited the Society by the remarkable sum of £12,607. The number of in-patients received at its Hospital at Hartshill was 256, the largest number yet recorded, but the number of out-patients has shown a falling off from the preceding year owing to the fact that a fewer number of discharged soldiers have been treated. The average cost of each in-patient per week was £2 11s. and of each out-patient attendance 3s. 0¼d., and the only notable increase in expenditure is upon salaries, which is partially accounted for by the staffing of country clinics. A generous offer of Biddulph Grange by Mr. Robert Heath was regretfully declined by the Committee, who felt that they could not foresee sufficient income for its annual maintenance as an Orthopædic Hospital.

Orthopædic Hospital, Hartshill: A Corner of the Massage Room.

## Figures and Tables

**Figure f1:**